# Effect of Chemical Attack Inhibitor Dosage on the Performance of Self-Compacting Concrete and Its Micro-Mechanisms

**DOI:** 10.3390/ma19132697

**Published:** 2026-06-23

**Authors:** Yuedong Wu, Jiaxiang Wang, Fangbin Zhang, Gen Li, Wen Lv, Rui Xu, Lei Zhang, Tianlei Wang

**Affiliations:** 1 State Key Laboratory of Iron and Steel Industry Environmental Protection, Central Research Institute of Building and Construction Co., Ltd., Beijing 102629, China; wuyuedong2015@163.com (Y.W.); 15766519011@163.com (F.Z.); viewgill@163.com (G.L.); lvwen10@126.com (W.L.); 2 Tianjin Key Laboratory of Building Green Functional Materials, School of Materials and Engineering, Tianjin Chengjian University, Tianjin 300384, China; 15612051222@163.com (J.W.); zhanglei@tcu.edu.cn (L.Z.)

**Keywords:** self-compacting concrete, chemical attack inhibitor, sulfate attack, chloride ion penetration, pore structure

## Abstract

Self-compacting concrete (SCC) is widely adopted in complex structural engineering due to its excellent flowability and filling capacity. However, in harsh corrosive environments, its complex internal pore structure can easily serve as a preferential pathway for the transport of aggressive media, leading to durability deterioration. This study systematically investigates the effects of chemical attack inhibitor (CAI) on the workability, mechanical properties, sulfate attack resistance, and chloride ion penetration resistance of SCC. The micro-mechanisms governing pore structure evolution are elucidated using low-field nuclear magnetic resonance (LF-NMR) and X-ray computed tomography (X-CT). At a CAI dosage of 2%, the fresh SCC exhibits a slump of 260 mm and slump flow of 720 mm, indicating excellent filling and gap-passing abilities. Meanwhile, the compressive strengths at 3 d, 7 d, and 28 d remain at a high level. After 120 sulfate wet-dry cycles, the strength loss rate is only 8.4%, with an erosion resistance coefficient exceeding 90%. In addition, the resistance to chloride ion penetration is significantly improved, with an electric flux of only 1331 C, which is considerably lower than that of the control group (1637 C). At the optimal dosage of CAI, the concrete exhibits a dense and uniform internal structure devoid of macroscopic defects or cracks, with minimized porosity, thus synergistically enhancing the resistance to sulfate attack and chloride attack. On the contrary, further increasing the CAI dosage markedly intensifies the inhibitory effect of organic components on cement hydration, leading to increased early-age defects and enhanced pore connectivity. Thus, an appropriate amount of CAI can effectively improve the overall performance of SCC, providing a solid experimental basis and theoretical support for its engineering application in harsh corrosive environments.

## 1. Introduction

Self-compacting concrete (SCC) is a typical special concrete that can flow and compact under its own weight without external vibration due to its excellent flowability, gap-passing ability, and segregation resistance, which has been widely used in engineering sections with dense reinforcement and complex structures [1]. In addition, the reduced reliance on mechanical vibration in SCC placement contributes to lower labor costs, enhanced construction quality, and improved operational safety [2]. Nevertheless, there are also limitations in the engineering application of SCC, including the high sensitivity of mix design to raw materials, the difficulty of quality control in the production process, and the strict adaptability requirements of admixtures and cementitious materials.

With the construction of engineering facilities gradually extending to harsh environments such as deep-sea areas, industrial corrosion areas, and high saline soil regions, the durability of SCC has attracted much attention as a key factor affecting its long-term service [3]. To achieve high fluidity and segregation resistance, SCC usually adopts a high paste-to-aggregate ratio. However, the compositional strategy results in a complex internal pore structure characterized by numerous connected pores and capillary channels [4,5]. Aggressive components can easily enter the concrete through capillary infiltration and diffusion with water as the carrier, which leads to the decomposition of hydration products, the deterioration of the interface transition zone, and the acceleration of steel corrosion, ultimately causing cracking, strength loss, and even structural failure [6]. Therefore, improving the durability of SCC in aggressive environments has become an urgent scientific and engineering problem.

Reducing the water-to-binder ratio can improve concrete compactness, thereby improving its corrosion resistance [7]. However, an excessively low water-to-binder ratio may exacerbate autogenous shrinkage and concentrate early-age hydration heat, increasing the risk of cracking [8,9]. Mineral admixtures can also improve corrosion resistance to some extent through pozzolanic and micro-filling effects, but they often exhibit slow early strength development and limited capacity to block connected pores [10,11,12]. The apparent chloride diffusion coefficient of SCC cured for 180 d in a high-salinity environment can be reduced by 50.83–54.40% compared with the reference group, which is attributed to the physical filling effect and pozzolanic activity of nano-silica that optimizes the interfacial transition zone and promotes the formation of C-S-H gel, thereby blocking chloride penetration channels [13]. Rahul et al. [14] demonstrated that metakaolin reduces mass fluctuations and compressive strength loss in SCC subjected to sulfate attack, with the sulfate resistance outperforming that of pure copper slag, reference, and combined silica fume groups. Zhang et al. [15] prepared concrete using a ternary mineral admixture of iron tailings powder, fly ash, and lithium slag as a cement replacement. When the cement replacement rate was 20%, and the water-to-binder ratio was 0.42, the acid corrosion resistance improved. In a simulated saline soil environment, ordinary slag exhibited better resistance than fly ash, while ultrafine slag achieved high durability under short-term steam curing, though its later-age performance improvement was inferior to that of ordinary slag [16]. The incorporation of limestone powder can not only reduce the chloride diffusion coefficient of SCC, but also improve slurry consistency and segregation resistance [17,18]. However, the protective effect of limestone powder against steel corrosion in a seawater environment is limited, and it needs to be used with corrosion inhibitors to achieve long-term protection [19]. Therefore, filling alone can only delay the ingress of the aggressive media into the matrix, and cannot fundamentally block the transport pathways at the source.

To overcome the shortcomings of traditional modification methods, researchers have begun exploring the application of organic hydrophobic admixtures and chemical erosion inhibitors in concrete. Cai et al. [20] studied the effect of internally mixed nano-hydrophobic admixture on the resistance to chloride corrosion. The admixture reduced the water absorption rate by more than 78% during capillary water uptake and impeded chloride ion transport. Liu et al. [21] prepared a hydrophobic mortar suitable for harsh engineering environments using ordinary Portland cement together with a hydrophobic agent. When the dosage of hydrophobic agent was 5%, the water absorption was reduced by 68.6%, the water loss rate was reduced by 27.3%, and both mass loss and linear expansion rates were greatly reduced, indicating that the hydrophobic agent can effectively slow water migration and reduce the sulfate attack. Gao et al. [22] developed a hydrophobic agent relatively stable in acid, alkali, and salt solutions, which can reduce water absorption and desorption rates of mortar and decrease the penetration of sulfate. Through hydrophobic interaction and pore structure refinement, the durability of mortar is improved. However, most existing studies focus on ordinary concrete, with systematic research on SCC lacking, and the multi-scale mechanism by which chemical erosion inhibitors regulate pore structure evolution remains unclear.

Based on the above, this study systematically investigates the influence of chemical attack inhibitor (CAI) on the workability, mechanical properties, and sulfate/chloride erosion resistance of SCC. By integrating LF-NMR and X-CT, the multi-scale regulation mechanism of CAI on pore structure is revealed. The findings are expected to provide a theoretical basis and experimental support for the engineering application of CAI in SCC serving in harsh corrosive environments, thereby promoting the continuous improvement of the long-term service performance of SCC.

## 2. Experimental Section

### 2.1. Materials

Ordinary Portland cement (P·I 52.5 grade) was produced by Qingdao Shanshui Innovation Cement Co., Ltd., (Qingdao, China). Its initial and final setting times are 188 min and 267 min, respectively. Class F fly ash (Grade I) was supplied by a power generation company in Qingdao, with an ignition loss of 0.84% and a specific surface area of 343 m^2^/kg. Ground granulated blast-furnace slag (S95 grade, a specific surface area of 415 m^2^/kg) was obtained from Qingdao Runyi Fengtai New Material Technology Co., Ltd., (Qingdao, China). The chemical compositions of the cementitious materials are presented in Table 1. Crushed basalt gravel with particle sizes of 5–10 mm and 10–15 mm was used as coarse aggregate. Fine aggregate was natural river sand (Zone II) sourced from Hebei Province. A polycarboxylate-based superplasticizer (PCA^®^-I) was manufactured by Jiangsu Subote New Materials Co., Ltd., (Nanjing, China), with a solid content of 20% and a water-reducing rate of approximately 28%. CAI was also produced by Jiangsu Subote New Materials Co., Ltd., (Nanjing, China). Its inorganic phase consists primarily of amorphous nano-silica, while the organic phase is composed of a polycarboxylic acid hydrophilic polymer. The main performance indicators are as follows: solid content of 15 wt%, density of 1.05 g/cm^3^ at 20 °C, pH of 7.5, primary particle size of nano-silica ranging from 15–40 nm, and emulsion dispersion particle size of 50–180 nm (D_50_ ≈ 90 nm).

### 2.2. Mix Proportion of Self-Compacting Concrete

The reference mix proportion of SCC is shown in Table 2, where the total amount of cementitious materials is 480 kg/m^3^, and the sand ratio is 40%. The cementitious material is composed of cement (264 kg/m^3^), fly ash (120 kg/m^3^), and slag powder (96 kg/m^3^). The aggregates are 720 kg/m^3^ of sand and 1080 kg/m^3^ of gravel, in which the mass ratio of 5–10 mm and 10–15 mm gravel is 0.15:0.85. CAI was incorporated by partially replacing the mixing water, at dosages of 2%, 3%, 4%, 5%, and 6% by mass of the cementitious material, and the corresponding test groups were designated CAI-2, CAI-3, CAI-4, CAI-5, and CAI-6, respectively.

### 2.3. Specimen Preparation and Curing

The gravel, sand, and part of the water were first placed into the mixer for 30 s. Subsequently, the cementitious material, remaining water, superplasticizer, and CAI were added, and mixing continued for 120 s. The mixer was then stopped for 90 s, followed by a final mixing stage of 60 s. The fresh mixture was immediately tested for workability and then cast into molds. After compaction on a vibrating table, the specimens were covered with plastic film to prevent water evaporation. They were kept at room temperature for 24 h before demolding and then transferred to a standard curing room maintained at 20 ± 2 °C and a relative humidity of ≥95% until the designated testing ages.

### 2.4. Characterization

The slump flow and J-ring flow of fresh concrete were tested in accordance with *Technical Specification for Application of Self-Compacting Concrete* (JGJ/T 283-2012) to evaluate filling ability and passing ability [23]. Compressive strengths of 100 mm × 100 mm × 100 mm cubic specimens cured for 3 d, 7 d, and 28 d were determined according to *Standard for Test Methods of Physical and Mechanical Properties of Concrete* (GB/T 50081-2019) [24]. Sulfate attack tests were carried out using the wet-dry cycling method specified in Standard for *Test Methods of Long-Term Properties and Durability of Ordinary Concrete* (GB/T 50082-2024) [25]. After 28 d of curing, specimens (100 mm × 100 mm × 100 mm) were subjected to cycles consisting of immersion in 5% Na_2_SO_4_ solution for 16 h, air-dried naturally for 1 h, dried at 80 °C for 6 h, and finally cooled for 1 h. Each complete cycle lasted 24 h. After 120 cycles, the compressive strength erosion resistance coefficient was measured to evaluate the sulfate resistance of concrete. According to GB/T 50082-2024, the rapid chloride ion permeability (electric flux) within 6 h was determined using cylindrical specimens (100 mm in diameter, 50 mm in height) cured for 28 d. Three parallel samples were used to test the compressive strength, electric flux, and sulfate attack performance, and the results were averaged. Low-field nuclear magnetic resonance (LF-NMR) was employed to analyze the pore water distribution and internal pore structure characteristics of concrete at different ages. Before testing, specimens were processed into cylinders of 20 mm in diameter and 20 mm in height, fully saturated, and then tested to obtain the transverse relaxation time (T_2_) spectrum measurements. The cube specimens (30 mm × 30 mm × 30 mm) were cast directly, solidified, and cured to 3 d, 7 d, 14 d, and 28 d for X-CT scanning. The internal pore structure was characterized by three-dimensional reconstruction and image processing.

## 3. Results and Discussion

### 3.1. Workability

**Figure 1:**
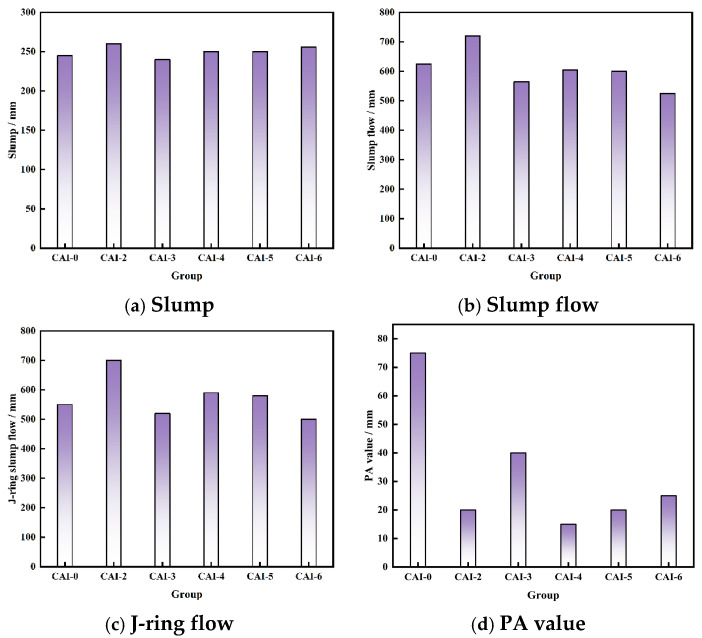
Influence of CAI dosage on workability of SCC.

Figure 1 shows the slump, slump flow, J-ring flow, and pass ability (PA) value of fresh concrete under different CAI dosages. As shown in Figure 1a, the slump of all mixtures is maintained between 240 mm and 260 mm through adjustment of the superplasticizer dosage, ensuring comparable basic fluidity across groups. The slump flow initially increases and then decreases with increasing CAI dosage (Figure 1b). When the dosage of CAI is 2%, the slump flow reaches the maximum value of 720 mm, which is significantly higher than that of CAI-0, indicating that the addition of an appropriate amount of CAI can effectively improve the fluidity of fresh concrete. The improvement is mainly due to the fact that the organic carboxylic ester polymer component in CAI has a certain water-reducing dispersion effect. Its adsorption on the surface of cement particles can reduce the frictional resistance between particles and release more free water to participate in the flow. However, when the CAI dosage exceeds 2%, the overall expansion degree shows a downward trend. The slump flow of CAI-6 is reduced to 525 mm, which is due to the fact that the equal-mass replacement of water by CAI reduces the free water dosage in the mixture. At the same time, the nanomaterials in CAI tend to agglomerate at higher dosages, increasing the viscosity of the cement paste and raising flow resistance. The trend of J-ring flow shown in Figure 1c is basically consistent with the trend of slump flow. The J-ring flow of CAI-0 is 550 mm, and that of CAI-2 is increased to 700 mm, indicating that an appropriate amount of CAI can significantly improve the gap-pass ability of SCC. As the dosage continues to increase, the J-ring flow gradually decreases, and the decrease is more obvious than that of slump flow. This indicates that under constrained conditions, the dual effects of CAI on the increase of slurry viscosity and the decrease of free water are more prominent, leading to a greater impairment of the concrete’s ability to pass through narrow gaps. It can be seen from Figure 1d that the PA value of CAI-0 is 75 mm. All CAI-containing groups exhibited significantly lower PA values, further confirming that CAI has a stronger impact on concrete fluidity under restricted flow conditions.

### 3.2. Compressive Strength

**Figure 2:**
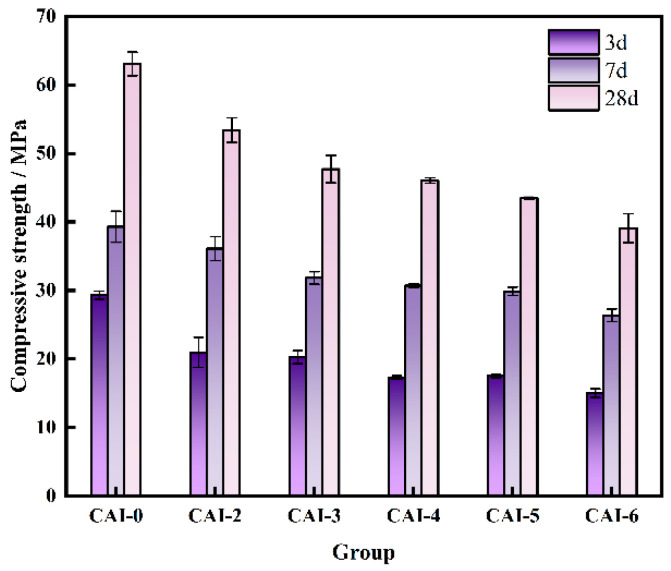
Influence of CAI dosage on compressive strength of SCC.

Figure 2 presents the effect of CAI dosage on the compressive strength of SCC at different curing ages. With the extension of the curing age, the compressive strength at different ages shows an upward trend, indicating progressive cement hydration and the gradual filling of pore space by hydration products. At the ages of 3 d, 7 d, and 28 d, the compressive strength of the test blocks shows a continuous downward trend with the increase of CAI dosage, indicating that the incorporation of CAI has an adverse effect on the mechanical properties. When the curing age is 3 d, the compressive strengths of all CAI-containing groups are significantly lower than that of CAI-0, highlighting a pronounced inhibitory effect of CAI on early-age cement hydration. This is mainly due to the adsorption of organic carboxylate polymers in CAI on the surface of cement particles to form a coating layer, which hinders the initial contact reaction between cement and water. In addition, the equal-mass replacement of water by CAI leads to a decrease in the actual free water dosage in the system, further delaying the hydration process [26]. At 7 d of curing, the compressive strengths of all CAI-containing groups are significantly lower than that of CAI-0. The strength gap between the reference group and the CAI groups narrows compared with that at 3 d, indicating that with the extension of curing age, the encapsulation effect of organic components in CAI on cement particles gradually decreases, and the inhibition effect on cement hydration gradually weakens. At the same time, with the continuous formation of hydration products, the ion concentration and pH value in the system increase, which further promotes the hydration reaction of unhydrated cement particles [27]. When the curing age is 28 d, the compressive strengths of CAI-containing specimens are still significantly lower than those of CAI-0. This is due to early-stage microstructural defects, such as loosely packed hydration products, which evolve into weak zones within the hardened paste. These defects cannot be fully compensated by later hydration, thereby restricting the full development of long-term strength.

### 3.3. Sulfate Attack Resistance

**Figure 3:**
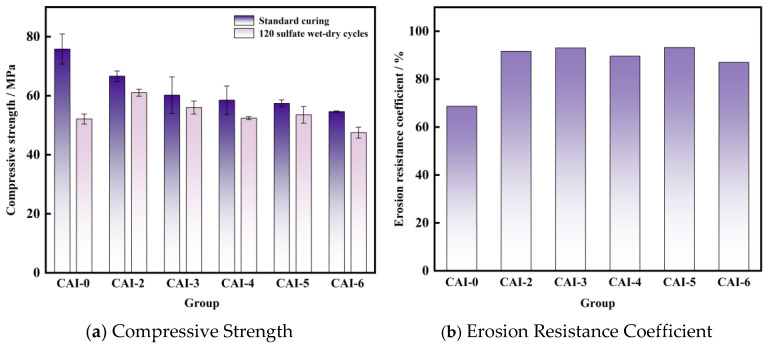
Influence of CAI on sulfate erosion resistance of SCC.

The compressive strength of SCC with different CAI dosages after 120 d standard curing and after exposure to sulfate dry-wet cycles is shown in Figure 3a. Under standard curing conditions, the compressive strength continues to decline with the increase of CAI dosage. The compressive strength of CAI-0 achieves the highest compressive strength of 75.8 MPa, whereas CAI-6 is only 54.6 MPa. After 120 sulfate dry-wet cycles, the compressive strength of CAI-0 decreases to 52.1 MPa, reflecting that the ordinary SCC suffers severe damage under sulfate attack. This deterioration is attributed to the reaction of sulfate ions with Ca(OH)_2_ and aluminate phases in cement hydration products, forming expansive products, which leads to the accumulation of internal expansion stresses that cause microcrack propagation and ultimately impair mechanical performance [28]. In contrast, all CAI-containing groups exhibit relatively smaller reductions in compressive strength after sulfate exposure, demonstrating that the addition of CAI effectively enhances the sulfate resistance of SCC. Notably, CAI-2 retains a high compressive strength after 120 cycles, with a strength loss rate of only 8.4%, which is significantly better than that of the CAI-0.

Figure 3b presents the erosion resistance coefficient of compressive strength with different CAI dosages after 120 sulfate dry-wet cycles. The CAI-0 has a coefficient of only 68.7% after 120 cycles, confirming that ordinary SCC is highly susceptible to deterioration under sulfate attack. In comparison, all CAI-containing groups exhibit coefficients exceeding 80%, with CAI-2 reaching over 90%. This marked improvement indicates that CAI significantly enhances the sulfate resistance of SCC. The organic carboxylic ester polymer in CAI forms a hydrophobic layer on the inner wall of pores and on the surface of hydration products, which significantly reduces water absorption, thereby blocking the contact between sulfate ions and the internal aluminum phase and Ca^2+^, inhibiting the formation of expansive products from the source [29,30,31]. At the same time, the organic components may consume part of the free Ca^2+^ through complexation, further reducing the thermodynamic driving force for erosion reaction. In addition, the nanomaterials in CAI can effectively fill the pores and improve the compactness of the matrix, thereby inhibiting the diffusion and transmission of sulfate ions.

### 3.4. Chloride Ion Permeability Resistance

**Figure 4:**
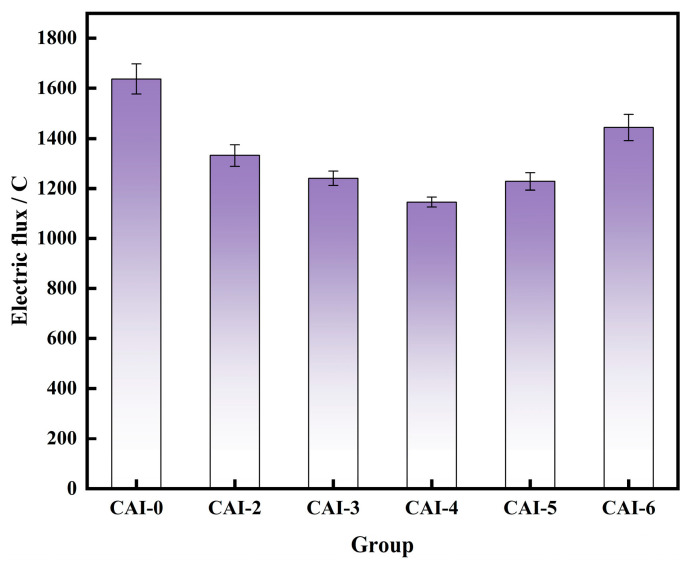
Influence of CAI dosage on electric flux of SCC.

As shown in Figure 4, the electric flux of CAI-0 is the highest at 1630 C, reflecting that the ordinary SCC has high chloride ion permeability. When CAI is added, the electric flux decreases, indicating that the addition of CAI can improve the resistance to chloride ion penetration. This can be attributed to the fact that the organic carboxylic ester polymer in CAI can form a hydrophobic film on the surface of cement hydration products and the inner wall of pores, which can significantly change the wettability and surface charge characteristics of pores, thereby increasing the tortuosity and resistance of directional migration of chloride ions under the action of an electric field [32]. At the same time, the nanomaterials in CAI can exert a micro-filling effect, filling in the pores and tiny voids and physically blocking ion transport pathways. However, with the increase of CAI, the electric flux decreases first and then increases, which is mainly due to the more serious influence of excessive CAI on the hydration reaction of cement, resulting in the gradual increase of its electric flux. Though the ion composition of the pore solution and the conductivity of the hydration product can affect the electric flux, the electric flux cannot be completely equal to the chloride ion migration rate in the real natural environment. However, this method is simple to operate and has a short test period, which can be effectively used for the relative comparison of chloride ion penetration resistance of concrete with different mix ratios.

### 3.5. Low-Field Nuclear Magnetic Resonance

**Figure 5:**
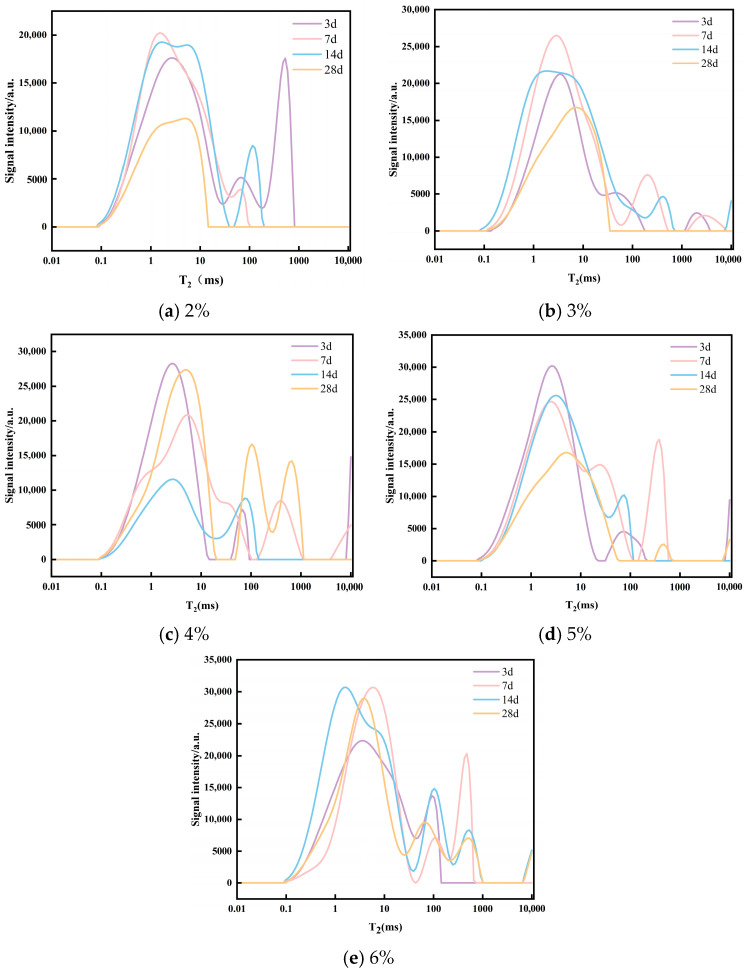
Low-field NMR spectra of SCC with different CAI dosages.

The T_2_ spectra obtained from LF-NMR of SCC with different CAI dosages at various curing ages are shown in Figure 5. The main peak of the T_2_ spectrum of each group is located in the region with relaxation time below 10 ms, indicating that the pore structure is predominantly composed of gel pores and fine capillary pores. With the exception of CAI-2, the other groups have obvious peaks in the relaxation time range exceeding 1000 ms, indicating that there are macro defects such as microcracks, large bubbles, or loose zones within the interfacial transition zone. The inhibition of organic components in CAI on the early-age hydration of cement leads to insufficient hydration products, which makes it difficult to effectively fill the initial accumulation gap. At the same time, the replacement of water by CAI increases the viscosity of the fresh paste, potentially hindering the release of entrapped air bubbles during mixing and leading to the retention of large pores. With the extension of curing age, the main peak of the T_2_ relaxation spectrum of the same CAI dosage group shows a significant left shift trend, and the peak area gradually decreases, reflecting the refinement effect of cement hydration on pore structure. At 3 d, the CAI-2 specimens display an additional peak in the range of 100–1000 ms, indicating that there are a certain number of mesopores in the specimens. With the extension of curing age to 7 d and 14 d, the mesopore peak gradually shifts leftward, and the peak intensity decreases synchronously, indicating that the mesopores are gradually filled or refined into smaller pores by hydration products. At 28 d, the T_2_ spectrum of the CAI-2 only retains a single main peak in the region where the relaxation time is less than 10 ms, corresponding to the inherent gel pores of the C-S-H gel. Macropores and mesopores inside the CAI-2 specimens have substantially disappeared, and the pore structure becomes dense and uniform. In contrast, for groups with higher CAI dosages, the leftward shift of the T_2_ spectra with increasing curing age is less pronounced, and additional peaks persist even at 28 d. This indicates that excessive CAI retards hydration to a greater extent, leading to a less refined pore structure with residual larger pores. Overall, as the hydration reaction advances, the continuous generation of hydration products fills the initial pore space, transforming the pore structure from coarse to fine and from interconnected to isolated, thereby improving the compactness of the hardened paste [33].

### 3.6. X-CT

**Figure 6:**
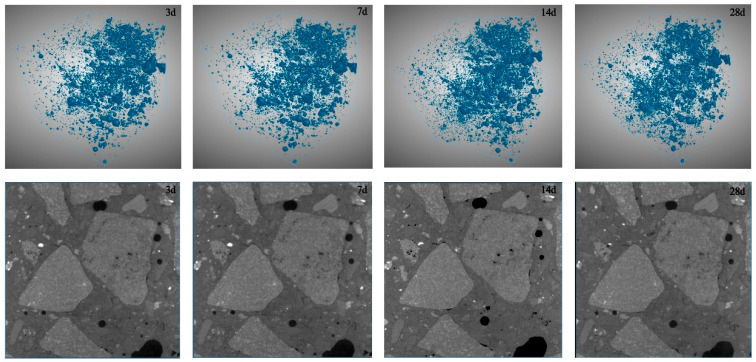
X-CT Image of SCC with 2% CAI dosage.

**Figure 7:**
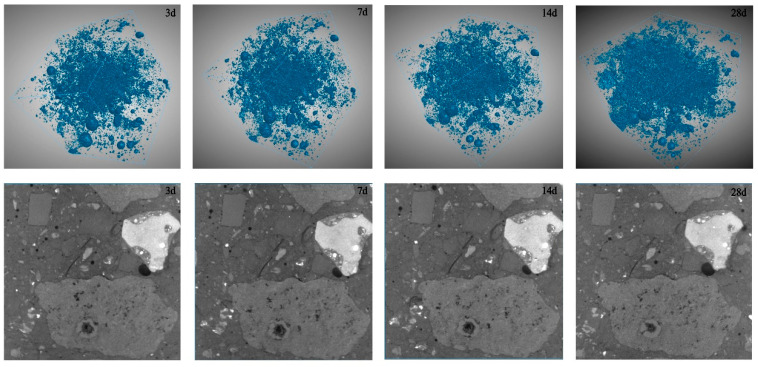
X-CT Image of SCC with 3% CAI dosage.

**Figure 8:**
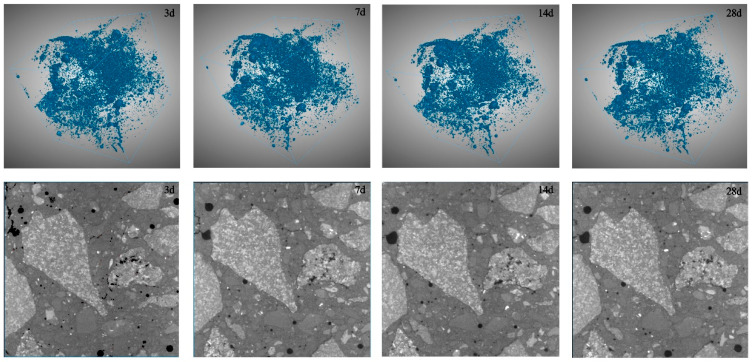
X-CT Image of SCC with 4% CAI dosage.

**Figure 9:**
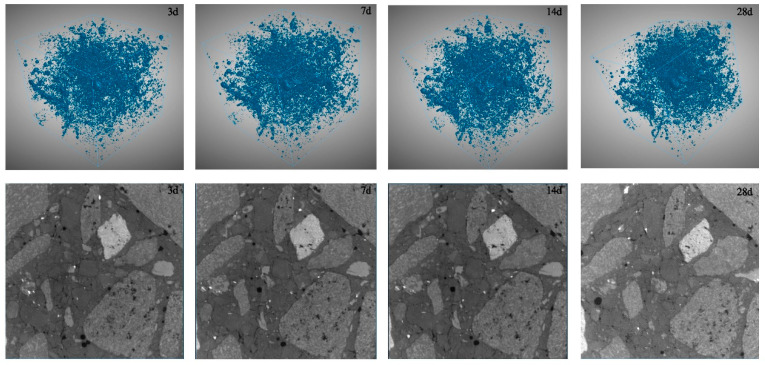
X-CT Image of SCC with 5% CAI dosage.

**Figure 10:**
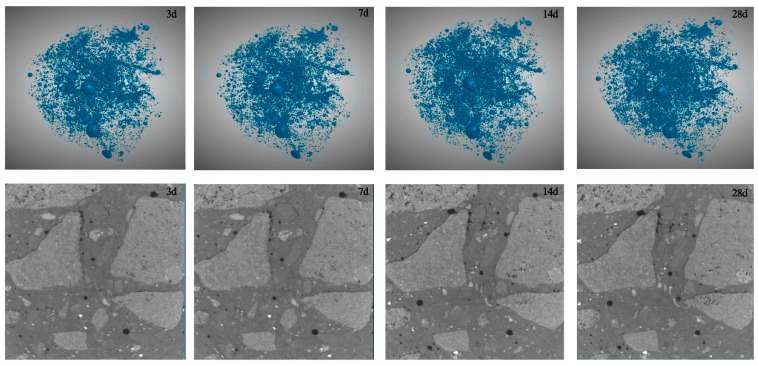
X-CT Image of SCC with 6% CAI dosage.

To further elucidate the spatial distribution characteristics of the internal pore structure of SCC with varying CAI dosages, X-CT was employed to perform three-dimensional scanning and reconstruction of hardened specimens at different curing ages (3 d, 7 d, and 28 d). The reconstructed images for CAI dosages of 2%, 3%, 4%, 5%, and 6% are shown in Figure 6, Figure 7, Figure 8, Figure 9 and Figure 10, respectively. A distinct relationship between CAI dosage and internal compactness, as well as a synergistic effect with curing age, was observed. Among all groups, the CAI-2 specimens exhibit the highest structural compactness at each curing age. At 3 d, only a few dispersed micropores can be observed, with no interconnected pores or microcracks. By 28 d, the internal pores have been continuously filled and refined by hydration products, resulting in a dense, homogeneous structure free of macroscopic defects or pore clustering. In contrast, the CAI-3 displays anomalous structural characteristics. At 3 d and 7 d, a large number of interconnected pores and microcracks are present, with disordered and aggregated pore distribution. When the CAI dosage is 3%, the organic carboxylic acid polymer is adsorbed to form a saturated, dense coating on the surface of the cement particles, which seriously delays early hydration, resulting in insufficient hydration products of the matrix and a sharp increase in pore connectivity. When the dosage exceeds 3%, the increase in the total amount of nano-silica makes its filling and pozzolanic effect gradually dominant, and the performance shows a relatively rebound. The dosage effect has been further discussed in the supplementary analysis.

**Figure 11:**
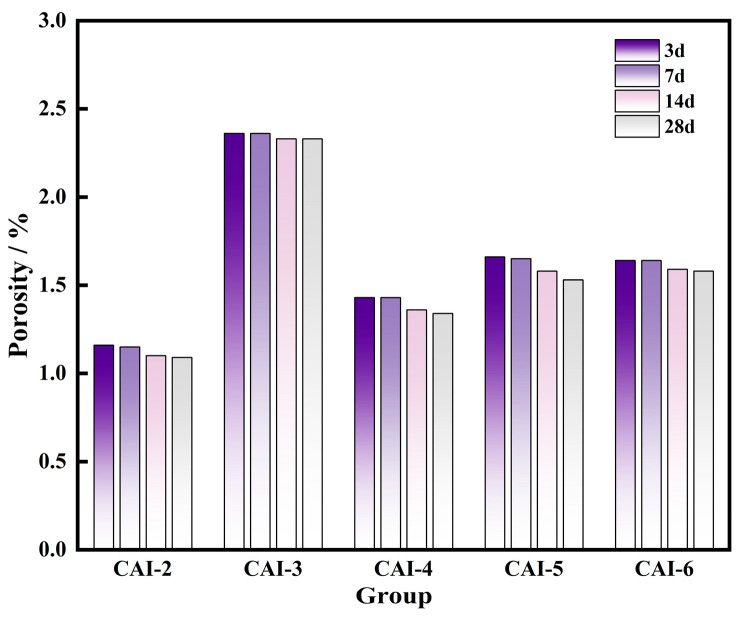
Porosity of SCC with different CAI dosages at various ages.

Figure 11 presents the porosity evolution of each group at different curing ages. Porosity decreases continuously with age for all mixtures, indicating ongoing pore filling by hydration products. At a CAI dosage of 2%, porosity drops from 1.16% at 3 d to 1.09% at 28 d. Although the reduction is modest, the absolute porosity remains the lowest among all groups at each age. When CAI dosage increases to 3%, porosity increases to over 2.2%, significantly higher than other groups at the same ages. Further increases in CAI dosage result in porosities of approximately 1.5%, which are still higher than that of the CAI-2 group. The abnormally high porosity of the 3% dosage group at all ages deserves special attention. This is likely due to a critical inhibition effect of CAI on cement hydration at this specific dosage, coupled with possible poor dispersion of the admixture, leading to the formation of numerous interconnected pores in the early stage that could not be fully refined later. In summary, at an optimal CAI dosage of 2%, the organic carboxylic ester polymer forms a hydrophobic film on hydration product surfaces, while the nanomaterials provide a micro-filling effect that refines the pore structure. Moreover, at this dosage, the inhibitory effect of CAI on cement hydration is weak, allowing continuous generation of hydration products. As curing progresses, the internal structure of concrete gradually becomes denser, thereby significantly improving resistance to both sulfate attack and chloride ion penetration. However, when the CAI dosage exceeds a certain threshold, the inhibitory effect becomes pronounced, leading to increased early-age defects and enhanced pore connectivity. Even with prolonged curing, the high porosity and pore connectivity cannot be fully compensated, resulting in degraded durability.

**Figure 12:**
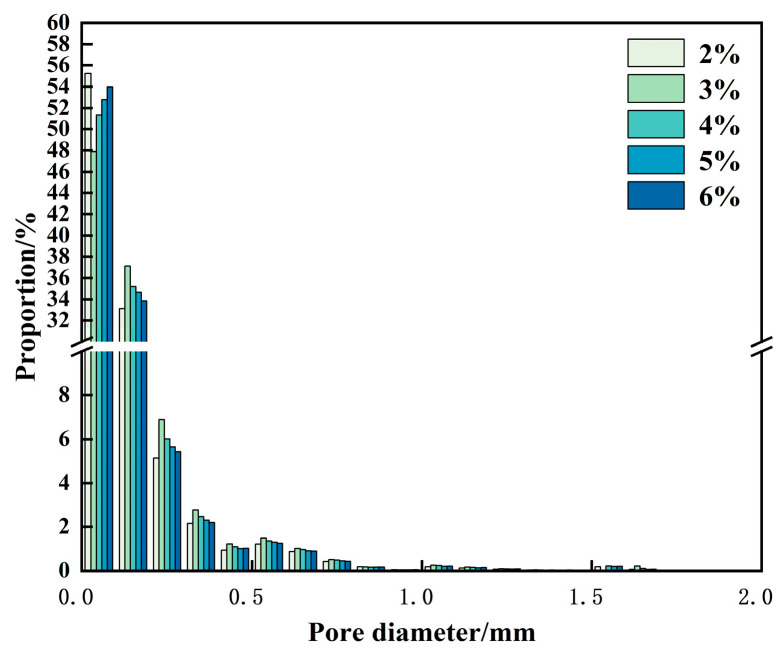
Pore distribution of SCC with different CAI dosages at 28 d.

Figure 12 presents the pore size distribution curves of SCC with different CAI dosages at 28 d. For all groups, pores are predominantly concentrated in the small pore range of 0–0.2 mm. Among them, the CAI-2 group exhibits the highest proportion of fine pores. The cumulative proportion of pores with diameters of 0–0.1 mm and 0.1–0.2 mm exceeds 88%, while the proportion of harmful mesopores and macropores is the lowest. In contrast, the CAI-3 group shows a marked decrease in the proportion of fine pores and a significant increase in harmful pores, confirming that this dosage exceeds the critical hydration inhibition threshold, leading to coarsening of the matrix pores. For CAI dosages of 4–6%, the proportion of fine pores gradually increases with increasing CAI dosage, indicating a certain improvement in pore structure, though the overall refinement remains inferior to that of the CAI-2 group. The pore size distribution is highly consistent with the macroscopic performance trends, demonstrating that refining the pore structure by reducing the proportion of harmful pores is the key mechanism by which CAI enhances the corrosion resistance of SCC. The triple synergistic effect of CAI can significantly refine the pore structure of SCC and reduce the proportion of harmful pores, thereby greatly improving its durability [34].

## 4. Conclusions

This study systematically investigated the effects of CAI on the workability, mechanical properties, sulfate attack resistance, and chloride ion penetration resistance of SCC. By combining LF-NMR and X-CT techniques, the multi-scale regulatory mechanism of CAI on pore structure evolution was revealed. When the CAI dosage was 2%, the slump flow and J-ring flow reached 720 mm and 700 mm, respectively, which were significantly higher than those of other groups. The addition of CAI reduced compressive strength at all curing ages due to the inhibitory effect of organic components on cement hydration and the reduction in free water dosage. However, CAI significantly enhanced the sulfate resistance of SCC. After 120 wet-dry cycles, CAI-2 exhibited a strength loss rate of only 8.4%, and the erosion resistance coefficient exceeded 90%, whereas CAI-0 suffered severe deterioration (coefficient 68.7%). At 2% CAI, the pore structure became dense and uniform, with mainly gel pores and fine capillary pores, and no macroscopic defects were observed at 28 d. Higher CAI dosages caused abnormal pore coarsening, increased connectivity, and persistent microcracks due to strong inhibition of hydration and insufficient hydration products. In summary, an appropriate amount of CAI improved the comprehensive performance of SCC by synergistically combining hydrophobic film formation and nano-filling, whereas excessive CAI impaired hydration and durability. This work provides a solid experimental basis and theoretical support for the engineering application of CAI in SCC exposed to aggressive environments.

## Figures and Tables

**Table 1:** Chemical composition of cementitious materials (wt%).

Oxide	Al_2_O_3_	CaO	Fe_2_O_3_	K_2_O	MgO	Na_2_O	SO_3_	SiO_2_	TiO_2_
P·I 52.5	4.20	63.63	3.89	0.38	2.15	0.34	2.05	17.37	0.26
Fly ash	41.56	4.16	4.93	0.67	0.42	0.012	0.78	43.45	1.85
Slag powder	17.51	39.63	0.34	0.37	7.43	0.33	2.04	31.0	0.61

**Table 2:** Mix proportion of SCC (kg/m^3^).

Group	Cement	Fly Ash	Slag Powder	Fine Aggregate	Coarse Aggregate	Water	CAI
CAI-0	264	120	96	720	1080	158	0
CAI-2	264	120	96	720	1080	148.4	9.6
CAI-3	264	120	96	720	1080	143.6	14.4
CAI-4	264	120	96	720	1080	138.8	19.2
CAI-5	264	120	96	720	1080	134	24.0
CAI-6	264	120	96	720	1080	129.2	28.8

## Data Availability

The original contributions presented in this study are included in the article. Further inquiries can be directed to the corresponding author.
